# Publication Recommendations to Report Laboratory Data of Neonates – a Modified Delphi Approach

**DOI:** 10.1038/s41390-024-03094-7

**Published:** 2024-03-06

**Authors:** Zoë Vander Elst, Heidrun Hildebrand, Mary A. Short, Nick Henscheid, Robert Ward, Ronald Ariagno, Thomas Diacovo, Tim Lang, Karel Allegaert, Anne Smits, The International Neonatal Consortium, Kanwaljit Singh, Carole Kenner, Deb Discenza, Hidefumi Nakamura, Jagdeep Podichetty, Jennifer Degl, Jonathan Davis, Mark Turner, Michael Padula, Satoshi Kusuda, Thierry Lacaze, Thomas Miller, Wakako Eklund, William Roddy

**Affiliations:** 1 Department of Development and Regeneration, KU Leuven, Leuven, Belgium; 2 Neonatal Intensive Care Unit, University Hospitals Leuven, Leuven, Belgium; 3 Bayer AG, Research & Development, Pharmaceuticals, Pediatric Development, Berlin, Germany; 4 International Neonatal Consortium, Communications Workgroup, Tucson, AZ USA; 5 Critical Path Institute, Tucson, AZ USA; 6 Department of Pediatrics, University of Utah, Salt Lake City, UT USA; 7 Department of Pediatrics, Division of Neonatal and Developmental Medicine, Stanford University, Stanford, CA USA; 8 Stony Brook University, Stony, Stony Brook, NY USA; 9 Blood Sciences, Royal Victoria Infirmary, Newcastle Upon Tyne Hospitals NHS Foundation Trust, Newcastle Upon Tyne, UK; 10 Committee of Emerging Technologies in Pediatric Laboratory Medicine, International Federation of Clinical Chemistry and Laboratory Medicine, Milan, Italy; 11 Department of Pharmaceutical and Pharmacological Sciences, KU Leuven, Leuven, Belgium; 12 Department of Hospital Pharmacy, Erasmus MC, Rotterdam, The Netherlands; 13 Council of International Neonatal Nurses, Inc. (COINN), Yardley, PA USA; 14 The College of New Jersey, Ewing, NJ USA; 15 PreemieWorld Foundation, Inc., Springfield, IL USA; 16 Alliance for Black NICU Families, Springfield, IL USA; 17 National Center for Child Health and Development, Tokyo, Japan; 18 Speaking for Moms & Babies, Inc., New York, NY USA; 19 NICU Parent Network, Georgetown, Washington, DC USA; 20 Tufts Medical Center, Boston, MA USA; 21 University of Liverpool, Liverpool, UK; 22 Children’s Hospital of Philadelphia, Philadelphia, PA USA; 23 Kyorin University, Tokyo, Japan; 24 Neonatal Research Network of Japan, Tokyo, Japan; 25 Maternal Infant Child and Youth Research Network, Vancouver, BC Canada; 26 University of Calgary, Calgary, AL Canada; 27 Bayer U.S. LLC, Whippany, NJ USA; 28 Pediatrix Medical Group of TN/National Association of Neonatal Nurses, Nashville, TN USA; 29 Pediatrix Medical Group of TN, Nashville, TN USA

## Abstract

**Background:**

Clinical and analytical information on laboratory data of neonates in scientific publications is sparse and incomplete. Furthermore, interpreting neonatal laboratory data can be complex due to their time-dependent and developmental physiology, and paucity of well-established age-appropriate reference ranges for neonates. This study aims to develop publication recommendations to report laboratory data of neonates to enhance the quality of these data in research and clinical care.

**Methods:**

A modified Delphi approach was used to develop recommendations in cooperation with the International Neonatal Consortium. A Core Group, including different stakeholders, was responsible for developing the recommendations, in collaboration with a Reflection Group, responsible for providing additional input.

**Results:**

The recommendations were classified into three categories: ‘Clinical Characteristics’, ‘Bio-analytical Information’ and ‘Data-analytical Information’. These were each divided into ‘Core Data’ (always to be reported) and ‘Supplemental Considerations’ (to be reported when considered relevant to the study).

**Conclusion:**

Our recommendations provide guidance on standardization of neonatal laboratory data in publications. This will enhance the comparison, replication, and application of study results in research initiatives and clinical practice. Furthermore, these recommendations also serve as foundational work to develop reference ranges for neonatal laboratory values by standardizing the quality of information needed for such efforts.

**Impact:**

- Standardized reporting of neonatal laboratory data in scientific publications will enhance the comparison, replication, and application of study results in research initiatives and clinical practice, as well as improve reporting to regulatory agencies.
- To integrate multistakeholder perspectives, a modified Delphi approach was used to develop publication recommendations which strengthens the applicability of the recommendations.
- Implementation of standardization will likely improve the overall quality of neonatal clinical research and neonatal healthcare.
- In addition, these recommendations are foundational to develop reference ranges for neonatal laboratory values by standardizing the quality of information needed for such efforts.

## Introduction

In scientific publications and clinical trials, laboratory data are collected and reported for various reasons, including the detection and quantification of effects and adverse events of treatments.^1^ Additionally, these values may serve as inclusion or exclusion criteria.^1^ Recently, the International Neonatal Consortium (INC)^2^ examined the quality of laboratory data reported in clinical studies of neonates.^1^ Published information on laboratory data of neonates proved to be sparse, not systematic and often incomplete.^1^ Furthermore, there appeared to be no specific standard for reporting laboratory data obtained from this population.^1^ Consequently, comparing and replicating published neonatal laboratory results, and applying this information in research initiatives or clinical practice remains problematic.

This information gap is part of a broader ‘replication and application crisis in clinical research’, emphasizing the need for generally accepted publication guidelines to report laboratory data.^3^ Being aware of this limitation that is not unique to neonates, the International Committee of Medical Journal Editors (ICMJE) has published general recommendations for the conduct, reporting, editing and publication of scholarly work in medical journals.^4^ They recommend describing methods, equipment and procedures in sufficient detail to allow replication of study results, as required by high impact science journals.^4^ In addition, they recommend providing references for established methods, describing any new methods used, explaining the reasons for using them, and evaluating their advantages and limitations.^4^ Other papers have also highlighted the importance of qualitative and structured reporting of study methods to enable the replication and application of study results in clinical practice.^3,5,6^

In neonates, interpreting laboratory data becomes even more complex due to their time-dependent and developmental physiology, the impact of maternal characteristics, and the paucity of well-established age-appropriate reference ranges.^1,7^ During the recent development of the Neonatal Adverse Event Severity Score, the absence of accepted reference ranges for neonatal laboratory data excluded these variables in the assessment of adverse events.^8^ To address this issue, the INC is currently working on defining normal and abnormal reference ranges for commonly used laboratory data in neonates.^9^ To obtain credible data for defining these reference ranges, a standardized method for collecting and reporting laboratory data of neonates is critical to improve data quality and utility.

Neonatal datasets are heterogeneous due to developmental changes occurring both before and after birth, as well as the effects of non-maturational factors like perinatal drug exposure.^7^ These distinctive population-specific characteristics necessitate precise and tailored recommendations for publications incorporating laboratory data of neonates, aimed at enhancing the quality of these data. These recommendations should align with established general guidelines for data-analytical and bio-analytical methods, while considering population-specific aspects such as neonatal clinical characteristics (e.g., gestational age, postnatal age, birth weight, nutrition), the use of low-volume samples, or clinical practices impacting laboratory values in both pre-analytical and analytical stages.

In this study, we aim to develop publication recommendations to report laboratory data of neonates using a modified Delphi approach to achieve more standardized reporting of these data. The Delphi approach is a structured communication method based on expert opinions and experience to reach consensus on specific research questions, and is commonly used to develop healthcare quality indicators.^10^ The implementation of the recommendations will support researchers and healthcare professionals with a standardized framework to report and interpret neonatal laboratory data in publications, thereby enhancing their applicability in clinical practice.

## Methods

### Modified Delphi approach

**Fig. 1:**
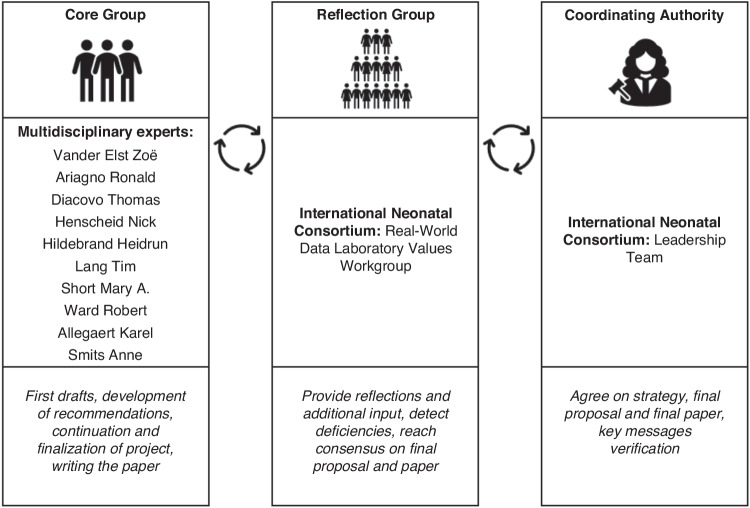
Modified Delphi approach. Overview of the modified Delphi approach used to develop publication recommendations to report laboratory data of neonates. A Core Group, consisting of 10 experts, was responsible for developing the recommendations and continuing the project. A Reflection Group, consisting of the International Neonatal Consortium’s Real-World Data Laboratory Values Workgroup, was responsible for providing additional input. The Coordinating Authority, consisting of the International Neonatal Consortium Leadership Team, was responsible for approving the project and final results.

Between November 2022 and August 2023, a modified Delphi approach was used to reach a stepwise consensus on recommendations to report laboratory data of neonates in scientific publications (Fig. 1). In a classic Delphi approach, participant answers are anonymized.^11^ Since we conducted online meetings, it was impossible to anonymize all answers. Hence, we used a modified Delphi approach. The recommendations were developed within the INC, a consortium of the Critical Path Institute. INC aims to advance neonatal regulatory science through coordinated efforts of different stakeholders.^12^ A Core Group, Reflection Group, and Coordinating Authority were key players during the development of the recommendations, as presented in Fig. 1. Online surveys and meetings were organized via commercially available software tools, and logistically supported by INC. We used a criterion of 80% agreement to move on to the next step. If 80% agreement was not achieved, an additional meeting was scheduled to further discuss the topic until consensus was reached.

### Stakeholder input

To integrate expertise from various perspectives, we included multidisciplinary stakeholders in the Core and Reflection Group (Fig. 1).

## Results

### Stakeholder representation

**Fig. 2:**
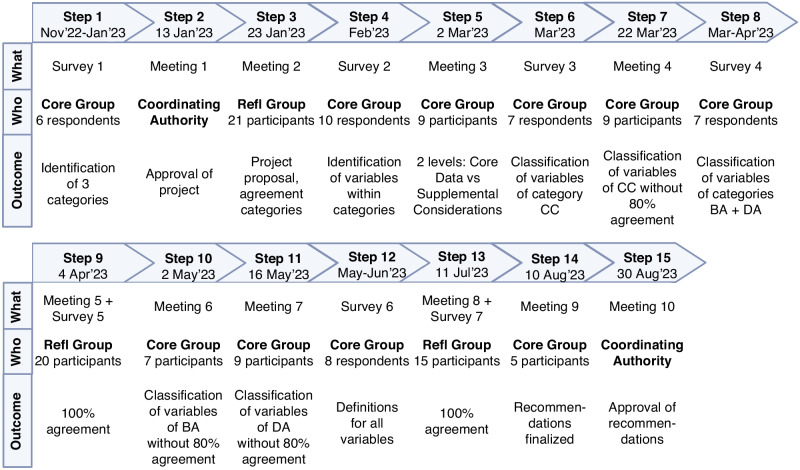
Workflow. Workflow of developing publication recommendations to report laboratory data of neonates. Refl Group Reflection Group, CC Clinical Characteristics, BA Bio-Analytical information, DA Data-Analytical information.

The Core Group consisted of ten experts (Fig. 1) with stakeholders from academia and industry, including clinicians, nurses, pharmacologists, doctoral and postdoctoral researchers, data analysts, and laboratory technicians, from Belgium, Germany, the Netherlands, the United Kingdom, and the United States of America (USA). In total, 16 additional experts participated in the Reflection Group with stakeholders from academia and regulators, including clinicians, pharmacologists, doctoral and postdoctoral researchers, data analysts, and laboratory technicians, from Canada, Europe, Japan, and the USA. The Coordinating Authority consisted of representatives from parents, nurses, academia, regulators, and industry. Participant numbers and outcomes for all consecutive steps are summarized in Fig. 2. All participants involved in at least one step are listed in the Acknowledgement section.

### Initializing the project and classifying the recommendations

Step 1: An online survey was sent to the Core Group to identify important categories within the recommendations (Fig. 2). Based on the responses of the Core Group members, the recommendations were divided into three main categories: 1) Clinical characteristics (CC), 2) Bio-analytical information (BA), 3) Data-analytical information (DA). First, clinical characteristics that could impact laboratory data should be considered. More specifically, this will enable interpretation and extrapolation of study results to other cohorts or care units with comparable characteristics. Second, specific bio-analytical information (information on laboratory analyses) is necessary for comparing, replicating, and applying the data provided in research initiatives and clinical practice. Third, data-analytical information (information on statistical analyses) is needed to assess the quality of the study and to understand how specific conclusions were derived.

Step 2: During an online meeting of the Coordinating Authority, the current project and workflow to develop publication recommendations to report laboratory data of neonates was proposed. The Coordinating Authority approved the project.

Step 3: An online meeting with the Reflection Group was organized to present the project and discuss the identified categories within the recommendations (Fig. 2). Overall agreement on the three proposed categories was achieved.

### Developing the recommendations

Step 4: A second online survey, sent to the Core Group, identified a first set of variables within the three categories.

Step 5: During an online meeting with the Core Group, variables within the category CC were discussed and agreed upon. To balance the need for information in publications to enable extrapolation of study results versus the burden of collecting this information, we decided to develop the recommendations on two levels for each of the categories. First, ‘Core Data’ with variables that should always be reported in publications regarding laboratory data of neonates, preferably in the methods or results section. Second, ‘Supplemental Considerations’ with variables that should be reported when considered relevant for the specific study. These supplemental variables may also be reported in appendices or supplementary materials. It is a set of variables that researchers should reflect upon when reporting laboratory data of neonates, but it is not an exhaustive list.

Step 6: In an online survey, the members of the Core Group classified the different variables within the category CC into ‘Core Data’ versus ‘Supplemental Considerations’. However, there were only 10 out of 74 variables with 80% agreement among the Core Group members (Supplementary Table S1).

Step 7: An additional online meeting with the Core Group was organized to discuss the variables within CC that did not reach the predefined criterion of 80% agreement (online survey of step 6) to classify them into ‘Core Data’ versus ‘Supplemental Considerations’. Overall agreement with the final classification was achieved.

Step 8: An online survey was set up for the members of the Core Group to classify the different variables within the categories BA and DA into ‘Core Data’ versus ‘Supplemental Considerations’. However, there were only 3 out of 48 variables with 80% agreement among the Core Group members (Supplementary Table S1).

Step 9: During an online meeting with the Reflection Group, the Core Group presented the category CC after which the Reflection Group was asked for additional input via an online survey: 100% agreement with this category was achieved.

Step 10: An additional online meeting with the Core Group was organized to discuss the variables within BA that did not reach the predefined criterion of 80% agreement (online survey of step 8) to classify them into ‘Core Data’ versus ‘Supplemental Considerations’. Overall agreement with the final classification was achieved.

Step 11: During a succeeding online meeting, the Core Group discussed the variables within DA that did not reach the predefined criterion of 80% agreement (online survey of step 8) to classify them into ‘Core Data’ versus ‘Supplemental Considerations’. Overall agreement with the final classification was achieved.

### Finalizing the recommendations

Step 12: Via an online survey between the Core Group members, definitions for all variables within the three categories of the recommendations were developed.

Step 13: During an online meeting with the Reflection Group, the Core Group presented the categories BA and DA after which the Reflection Group was asked for additional input via an online survey: 100% agreement with these categories was achieved.

Step 14: A final online meeting with the Core Group was organized to finalize the recommendations. The final version of these recommendations is presented in Table 1 (Core Data) and Table 2 (Supplemental Considerations).

**Table 1:** Publication recommendations to report laboratory data of neonates – Core Data.

**Core Data**^a^	
*Categories and variables*	*Definitions and/or examples*
**Clinical Characteristics**^b^	
Study population description	
Country	*Country or countries where the study was performed*
Number of neonates	*Number of neonates included in the study that contributed laboratory data*
Sex	*Male/female/undetermined*
Race and ethnicity	*Report which classification was used and who identified patient race and ethnicity (e.g. self-reported, investigator observed, database retrieved…), be specific and consistent*
Gestational age	*Number of weeks from the first day of the mother’s last menstrual cycle to birth*
Postnatal age	*Chronological age after birth, provide a definition of day of birth (day 0 or day 1)*
Birth weight	*Weight of the newborn at birth*
Relevant diagnoses	*Report whether neonates with these specific diagnoses were included in the study or not*
Severe infections/sepsis	*E.g. pneumonia, early or late onset sepsis…*
Kidney disorders	*E.g. acute kidney injury, congenital anomalies of the kidney and urinary tract…*
Hematological diseases	*E.g. hemolytic anemia, neonatal alloimmune thrombocytopenia…*
Necrotizing enterocolitis	*Report Bell staging criteria*
Hypoxic-ischemic encephalopathy	
Relevant treatment modalities	*Treatment given around or during laboratory tests*
Blood product transfusions	*E.g. packed cells, fresh frozen plasma, platelets…*
Phototherapy	
Indication for laboratory testing	
Screening	*E.g. newborn bloodspot screening, screening for potential conditions…*
Diagnosis	
Monitoring/follow-up	
Study purpose	*E.g. clinical trial*
**Bio-analytical Information**^c^	
Sample information	
Type of sample	*E.g. arterial/venous/capillary, full blood/serum/plasma, dried blood spot, urine, cerebrospinal fluid, pleural or peritoneal fluid*
Sample storage	*Temperature at which the sample was stored and possible failure to store it correctly*
Tube details	*Preservative of the tube in which the sample was taken*
Laboratory information	
Name and address	*Name and address of the laboratory where the tests were performed*
Analysis methods	
Assay	*Name of assay and specific assay used, based on ultra-low blood volumes*
Manufacturer	*Manufacturer of the assay used*
Equipment	*Analyzer the assay is run on*
Change of methods	*Any changes in bio-analytical methods used during the study period*
References of methods	*Refer to the methods used, a reference should describe the performance of the assay*
Traceability	*Traceability to an international standard yes/no*
Reference ranges or decision limits used and rationale for using them	*Reference ranges are the normal range for a specific laboratory value, decision limits are levels when an action must be taken to prevent harm or to initiate a management. Report supporting literature and information on source population*.
Units of measurement	*Including conversion factors of units*
**Data-analytical Information**^d^	
Data collection	
Population size considerations	*Number of observations and statistical power calculation*
Data storage format	*E.g. SDTM (Study Data Tabulation Model), OMOP (Observational Medical Outcomes Partnership)…*
Data processing	
Statistical software used	*Software used to process the data*
Data cleaning	
Quality of data	*Including bias, coding issues, reporting errors, quality control checks…*
Incomplete/missing data and methods used to handle them	*E.g. deletion, imputation…*
Handling of duplicates	*Method to identify and remove duplicates*
Handling of values below LLOQ/LLOD	*LLOQ; lower limit of quantification, LLOD; lower limit of detection. E.g. imputation with interval regression, substitution with LLOD/2, deletion…*
Data analysis	
Software used	*Software used to analyze the data*
Formulas/algorithms used	*Statistical tests used to analyze the data*
Decision limits and rationale	*Statistical decision limits, e.g. significance level*
Reporting results	*Appropriate measures for statistical significance, e.g. p-values, confidence intervals…*

^a^‘Core data’ should always be reported in publications regarding laboratory data of neonates, preferably in the methods or results section of the publication.

^b^Clinical characteristics that could impact laboratory data should be considered.

^c^Specific bio-analytical information is necessary for comparing, replicating, and applying the data provided in research initiatives and clinical practice.

^d^Data-analytical information is needed to assess the quality of the study and to understand how specific conclusions were derived.

**Table 2:** Publication recommendations to report laboratory data of neonates – Supplemental Considerations.

**Supplemental Considerations**^a^	
*Categories*	*Definitions and/or examples*
**Clinical Characteristics**^b^	
Study population description	*E.g. postmenstrual age, current weight, growth charts used, small/large for gestational age, multiple pregnancy…*
Details related to the delivery	*E.g. vaginal delivery, caesarean section, primary/secondary/urgent caesarean section, cardiotocogram: fetal distress, APGAR score at 1/5/10* *minutes, resuscitation yes/no…*
Maternal characteristics	*The neonatal period varies from 28 days in a term infant to months for a very premature infant; therefore, reporting maternal information for impact on neonatal laboratory values may be dependent on the postnatal age of the infant. E.g. GBS status, medication (ab)use during pregnancy, illegal drug use during pregnancy, folate supplementation, concurrent diagnoses, diagnoses related to pregnancy (e.g. gestational diabetes mellitus), infections during pregnancy, chorio-amnionitis, diet during pregnancy, weight gain and BMI during pregnancy…*
Prenatal medication/interventions	*Specify medication class and name. E.g. corticosteroids for lung maturations, maternal antibiotics, fetal interventions…*
Postnatal medication	*E.g. antibiotics, non-steroidal anti-inflammatory drugs, analgesics, sedatives, surfactant…*
Standard practices within the neonatal unit	*E.g. delayed cord clamping, standard prophylactic medication, standard intravenous fluids and parenteral nutrition, lipid supplementation, standard antibiotic regimens, feeding practices…*
Relevant diagnoses	*E.g. respiratory distress syndrome, pulmonary interstitial emphysema, bronchopulmonary disease, intraventricular hemorrhage, periventricular leukomalacia, seizures, patent ductus arteriosus…*
Relevant treatment modalities	*E.g. therapeutic hypothermia, extracorporeal membrane oxygenation, renal replacement therapy, respiratory support and ventilation type, phototherapy thresholds, use of intensive phototherapy, vitamin supplementation…*
**Bio-analytical Information**^c^	
Sample information	*E.g. how the sample was transported, manufacturer of the tube in which the sample was taken, quality of the sample (hemolytic/clotted), hemolysis/lipemic/icteric index…*
Date	*Date when the sample was taken and time between taking the sample and the test being performed*
Laboratory information	*E.g. performance specifications of the laboratory, accreditation of the laboratory (national accreditation status against ISO 15189 standards), external quality assessment…*
Analysis methods	*E.g. batch number of the assay used, calibration methods used, centrifugation details, FDA approved yes/no, traceability of the method used to which international standard, intra- and inter-assay variability, if new methods used; rationale for using them and validation efforts, interferences with methods, are tests repeated if outside of physiological range, was stability tested in the local storage conditions…*
Analytical performance of the test	*E.g. sensitivity, specificity, positive/negative predictive value, lower limit of quantification, accuracy of the test…*
**Data-analytical Information**^d^	
Data collection	*E.g. procedures for data verification; how to handle results when out of ‘normal’ range, compliance with predetermined analytical plan…*
Data access statement	*Data available for public access yes/no, if yes; how to access them*

^a^‘Supplemental considerations’ should be reported when considered relevant for the specific study (in appendices or supplementary materials). It is a set of variables that researchers should reflect upon when reporting laboratory data of neonates, but it is not an exhaustive list.

^b^Clinical characteristics that could impact laboratory data should be considered.

^c^Specific bio-analytical information is necessary for comparing, replicating, and applying the data provided in research initiatives and clinical practice.

^d^Data-analytical information is needed to assess the quality of the study and to understand how specific conclusions were derived.

### Approval by the coordinating authority

Step 15: During an online meeting, the final recommendations were presented to the Coordinating Authority. The Coordinating Authority approved the recommendations.

## Discussion

To enhance the quality and utility of published laboratory data of neonates, we developed recommendations on how to report these data. The recommendations were classified into three categories: ‘Clinical Characteristics’, ‘Bio-analytical Information’ and ‘Data-analytical Information’. These were each classified into ‘Core Data’ and ‘Supplemental Considerations’. The recommendations are non-binding, but should provide a framework for researchers when reporting laboratory data of neonates in scientific publications. The recommendations are intended to be consulted before data collection takes place to ensure structured data collection which is a key step in standardization of data reporting.

As described in the results, multiple discussions were needed to reach consensus on the recommendations. ‘Clinical Characteristics’ was the most challenging category to achieve the appropriate balance between data needed to interpret laboratory values of neonates versus the burden of collecting and reporting all these data. The compromise led to the creation of ‘Core Data’ and ‘Supplemental Considerations’. ‘Core Data’ are necessary to interpret and potentially extrapolate study results. Therefore, depending on the study, components of ‘Supplemental Considerations’ could be considered as core information. Authors, reviewers, and editors are encouraged to evaluate the need for reporting variables in ‘Supplemental Considerations’ to enable the interpretation and extrapolation of study results.

The ICMJE has already published general recommendations to report scholarly work on laboratory data in medical journals.^4^ However, these recommendations mainly focus on the bio-analytical method section of papers and are not specific to neonates. Innovative in our recommendations is the multistakeholder approach and its focus on a special patient population requiring specifications related to clinical characteristics (e.g., gestational age, postnatal age, birth weight, neonatal co-morbidities, maternal characteristics) and bio-analytical information (e.g., low-volume samples).

Specific age-appropriate reference ranges for many laboratory values are currently missing for neonates. Well-established reference ranges are important for interpreting neonatal laboratory results, and will improve the overall quality and precision of neonatal medicine. Our recommendations are foundational work to standardize the quality of information needed for developing such reference ranges.

Multiple stakeholders will benefit from a more standardized approach of reporting neonatal laboratory data in scientific publications. To obtain multistakeholder perspectives, the INC Communications Workgroup provided input from physicians, nurses, parents, industry, and regulators. Physicians will mostly benefit from a more standardized reporting of neonatal clinical characteristics to enable extrapolation of study results to their patient population. More standardized reporting of neonatal laboratory data will improve the value of these data for the scientific community which is considered as important by both nurses and parents, since collecting samples to obtain these results can be burdensome. This might even increase parent’s trust in neonatal clinical research, potentially increasing the percentage of parental consent to enroll their children in clinical trials. From an industry perspective, enhanced comparison, reproducibility, and application of study results is perceived as most important since this may increase the overall quality of neonatal clinical research. Regulators prioritize the ability to timely refer Sponsors to our newly developed recommendations to guarantee appropriate clinical trial design and the collection of comprehensive information right from the study’s onset.

There are some limitations to the developed recommendations. First, we chose to include race and ethnicity as a variable in the category CC. However, race- and ethnicity-based medicine is a controversial topic in clinical practice, medical literature and research, and often displays collinearity with other covariates of interest.^13^ Both race and ethnicity are often used interchangeably and there is no generally accepted definition for these covariates. Most historical definitions of race are based on ancestral origin and physical characteristics, and of ethnicity on cultural identity.^14^ Race and ethnicity are often included in medical studies and treatment decisions to account for biological or genetic differences, and to ensure representativeness, as these may impact clinical outcomes. For example, race-based corrections are made for estimating glomerular filtration rate.^15^ However, both race and ethnicity are social constructs without biological or genetic meaning.^14,16^ The way in which these characteristics are collected differs between studies, e.g. it can be self-reported, assigned by the investigator, or retrieved from a database or from electronic health records.^14^ Often, differences allocated to a specific race or ethnicity can be explained by other factors such as diet, sun exposure, demographics, social determinants, etc.^13,14^ The guidelines reported by Bakkum et al. do recommend to report race and ethnicity, but encourage sponsors, researchers and clinicians to be aware of how these data were collected, and they advise to be cautious in drawing conclusions solely based on race.^13^ Recent guidelines on how to report race and ethnicity in medical and science journals from the Journal of the American Medical Association advice to report race and ethnicity in a specific and consistent way by using subcategories, to report which classification was used, to report who identified race and ethnicity, and to explain the reason for assessing race and ethnicity.^14^

Second, we did not advise on how to report units for each laboratory value. However, a more uniform way of reporting units of laboratory values would very likely add to the generalization and extrapolation of study results.^17^ The Unified Code for Units of Measure (UCUM) is a universal coding system that includes all currently used units of laboratory values to facilitate communication in research.^18^ We strongly recommend the use of this system when reporting units of laboratory values. Additionally, harmonizing terminologies for laboratory tests and diseases will facilitate communication among researchers, as well as among patients and regulatory agencies. For laboratory tests, LOINC (Logical Observation Identifiers Names and Codes) is a common language to identify health measurements, observations, and documents.^19^ It describes the different components of the sample, analysis, units, and methods into a single term. When the source data includes LOINC codes, we recommend including these along with the description of the assay. Similarly, there are a number of terminologies that describe diseases in a systematic way. Some common disease terminologies are SNOMED-CT (Systemized Nomenclature of Medicine Clinical Terms), ICD (International Classification of Diseases), and MedDRA (Medical Dictionary for Regulatory Activities).^17^ If available, we encourage the inclusion of these codes to identify diseases in publications.

Third, the Delphi approach also has its limitations. Its time-consuming nature and reliance on expert opinions may introduce participant bias. However, when conducted with a structured and well-organized framework, this approach can yield valuable and reliable results. Recognizing the importance of diverse perspectives, we included stakeholders with various scientific, professional, and geographical backgrounds, thereby ensuring a comprehensive and multidisciplinary expertise panel. Through this approach, we aimed to mitigate the possible limitations of a Delphi approach, thereby improving the credibility of our results.

## Conclusion

Publication recommendations to report laboratory data of neonates were developed. These recommendations apply to neonates, a special patient population requiring specific recommendations. The recommendations are based on three categories: ‘Clinical Characteristics’, ‘Bio-analytical Information’ and ‘Data-analytical Information’. Each of these categories is classified into ‘Core Data’ (variables that should always be reported) and ‘Supplemental Considerations’ (variables that should be reported when considered relevant to the study). The implementation of these recommendations will enhance the comparison, replication, and application of study results in research initiatives and clinical practice. This is likely to significantly contribute to the overall quality of neonatal clinical research and neonatal healthcare. In addition, this is foundational work for developing reference ranges for neonatal laboratory values by standardizing the quality of information needed for such efforts.

### Supplementary information

Supplementary Material

## Data Availability

The corresponding author can be contacted to share the raw data, if based on a reasonable request and study protocol.
